# Liquid-crystalline nanoparticles: Hybrid design and mesophase structures

**DOI:** 10.3762/bjoc.8.39

**Published:** 2012-03-08

**Authors:** Gareth L Nealon, Romain Greget, Cristina Dominguez, Zsuzsanna T Nagy, Daniel Guillon, Jean-Louis Gallani, Bertrand Donnio

**Affiliations:** 1 Institut de Physique et Chimie des Matériaux de Strasbourg (IPCMS), CNRS-Université de Strasbourg (UMR 7504), 23 rue du Loess, BP 43, 67034 Strasbourg Cedex 2, France

**Keywords:** hybrid, liquid crystal, mesogen, mesomorphism, mesophase, nanoparticle, self-organisation, supramolecule

## Abstract

Liquid-crystalline nanoparticles represent an exciting class of new materials for a variety of potential applications. By combining supramolecular ordering with the fluid properties of the liquid-crystalline state, these materials offer the possibility to organise nanoparticles into addressable 2-D and 3-D arrangements exhibiting high processability and self-healing properties. Herein, we review the developments in the field of discrete thermotropic liquid-crystalline nanoparticle hybrids, with special emphasis on the relationship between the nanoparticle morphology and the nature of the organic ligand coating and their resulting phase behaviour. Mechanisms proposed to explain the supramolecular organisation of the mesogens within the liquid-crystalline phases are discussed.

## Introduction

The term "nanotechnology" has gone far beyond the realms of universities and research laboratories and is understood by scientist and layman alike to be synonymous with "high-tech" and "futuristic", promising to bring about tremendous advances in our quality of life. By utilising a "bottom-up" approach, whereby atomic and molecular components are assembled in a controlled fashion [1], scientists hope to greatly expand the scope of accessible technologies and tackle the existing and future challenges in novel ways. Perhaps the most ambitious goal of this field of endeavour is the creation of molecular machines and devices, with dimensions on the order of atoms and molecules, capable of performing specific predetermined tasks [2]. At the core of this nanotechnology revolution lies the nanoparticle (NP), the most common of which being metallic particles (including alloys and oxides) with at least one dimension smaller than 100 nm. These species can exhibit physical and chemical properties that differ tremendously from their bulk counterparts, and which depend not only on their composition but also on their shape and size [3].

Nanoparticle science is ever expanding with its interest derived from the desire to understand the synthesis and fundamental properties [4–9] and the myriad of potential applications of these unique species [10]. Fields as diverse as biology and medicine [11–12], optics and electro-optics [13–14], catalysis [12,15], and environmental remediation [16] are expected to benefit from the unique properties and promises offered by nanoparticles. Of course, in order to exploit the exciting properties of NPs, it is almost always necessary to incorporate them into a more complex structure, often through the use of a suitable organic coating, which not only imparts stability against aggregation but also other important features such as solubility, modularity, and optical and self-organisation properties.

The organisation of NPs into ordered systems is of crucial importance for their use in high-technology applications and devices, and a wide variety of "top-down" and "bottom-up" methods have been proposed and are continuously under development [17–20]. Central to these efforts is a consideration of the forces acting upon the NPs [21–22], which are determined by their composition (surface chemistry, magnetic/electrostatic properties) and morphology (size, shape and roughness) and can be manipulated by coating with suitable structure-directing agents. Amongst the techniques used to assemble NPs, self-assembly methods show immense promise towards achieving the ambitious results expected from the field of nanotechnology research [23]. Liquid-crystalline (LC) materials offer unique opportunities in the field of NP organisation due to the intrinsic order and fluid properties that they possess, allowing for complex architectures to be established with high processability and defect tolerance. The properties of LCs can also be influenced by external factors, such as applied magnetic and electric fields and surface effects, all of which lend these materials towards their use in stimuli-responsive advanced materials [24]. What follows is a brief introduction to some of the salient points in LC science, but a thorough introduction to the theory and background of liquid crystals is beyond the scope of this review, and the interested reader is directed to numerous texts on the subject [25–29].

The liquid-crystalline state is intermediate between that of a perfectly ordered crystal and a disordered isotropic liquid. The moieties within a liquid crystal possess intrinsic directional order, often accompanied by various degrees of positional order, whilst remaining in a fluid state. A material that exhibits a liquid-crystalline state as a function of temperature is referred to as *thermotropic*, whereas those that exhibit a LC phase in the presence of a solvent are known as *lyotropic*. A material that exhibits LC properties is referred to as a *mesogen* and is said to exhibit *mesomorphism*; although something that is *mesogenic* (e.g., proto-mesogens) is not necessarily *mesomorphic*.

**Figure 1:**
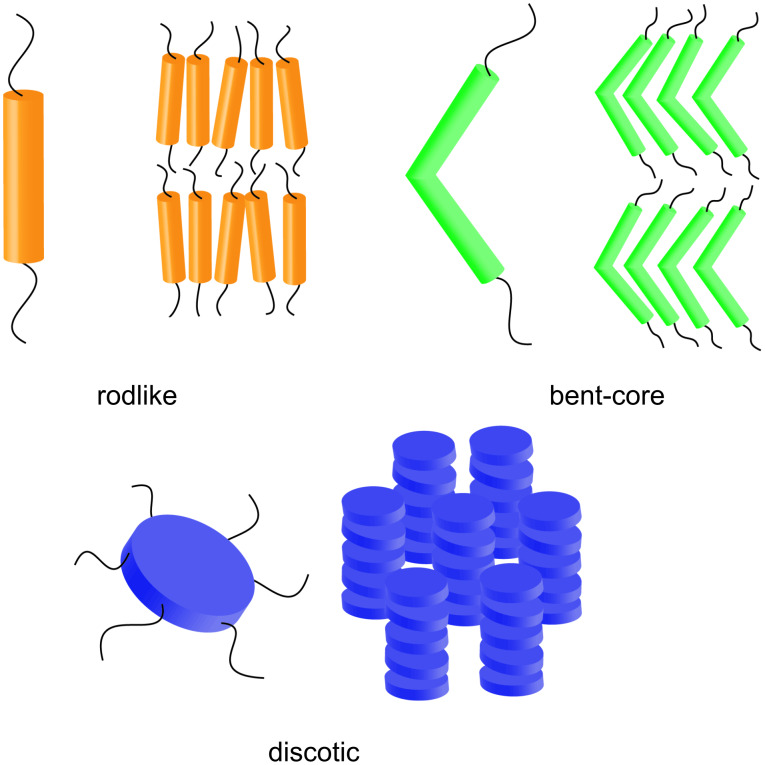
Three of the common molecular and supramolecular structural motifs in liquid crystal chemistry: rodlike, bent-core and discotic mesogens.

Thermotropic liquid crystals are usually formed by molecules that contain at least two sections with contrasting chemical or structural character (*amphipathic*), most often in the form of a rigid anisotropic moiety appended with flexible segments. It is this amphipathic nature of the molecular structure that brings about the multi-step melting process that is characteristic of the liquid-crystalline state, due to the phase separation of the incompatible components [24,30–33]. Since the anisotropy of the molecular structure plays such an important role in the formation and properties of liquid-crystalline phases, the types of ligands involved are often classified according to their general shape characteristics. Thus, rodlike or calamitic molecules, for example, possess one axis much longer than the others, whereas discotic molecules exhibit one axis much shorter than the other two (Figure 1).

There are a variety of liquid-crystalline phases possible, based on the shape and chemical characteristics of the mesogen under investigation. The simplest is the nematic phase, whereby the mesogenic moieties exhibit an average alignment along a common direction (or *director*), whereas the smectic phases also display a degree of positional ordering (i.e., the mesogens with essentially calamitic structures are arranged into layers). Columnar phases arise from the stacking of disclike mesogenic moieties into columns, which are in turn often arranged in a parallel manner into 2-D ordered lattices. Other mesophases include those possessing 3-D symmetry (cubic or tetragonal), and the so-called banana phases formed by bent-core mesogens [30,34–35].

This review will focus on publications that describe the preparation and characterisation of discrete thermotropic liquid-crystalline nanoparticle hybrids. Thus topics such as micellar [36] or lyotropic NPs [37–38], polymer coated/embedded NP mesogens [39–41] and the expansive field of NP-doped LC systems (including labile hybrid/ligand mixtures [42] and direct synthesis of NPs in LC matrices [43–46]) of interest for various high-technology applications [47], will not be discussed here, but the interested reader is directed to other publications in the field [48–49].

This review is structured in such a way as to highlight the various methods that have been successfully, and unsuccessfully, utilised in the quest for the preparation of nanoparticulate thermotropic liquid-crystalline materials. The various materials are classified according to the type of ligand coating used to impart mesogenic behaviour to the hybrid, highlighting their pivotal role in the design process. It is hoped that the reader will gain an understanding of the various factors that influence the mesophase behaviour of these new materials, and to appreciate some of the early trends that are appearing in this relatively young and exciting field of endeavour.

## Review

### Design considerations

In order for a nanoparticle sample to exhibit a thermotropic liquid-crystalline phase, it is necessary to first coat the NPs with a suitable material that allows, at the very least, the particles to form a fluid phase at relatively moderate temperatures (i.e., ideally <250 °C). From here, it becomes apparent that if one considers the two most commonly encountered NP morphologies, that is, pseudospherical polyhedra and anisotropic rod-/needlelike and platelike shapes, different strategies may be possible for inducing mesophase behaviour in these hybrids.

In the case of rod-/needlelike NPs with high aspect ratios, their inherent shape lends itself to the formation of LC phases such as nematics and smectics, subject to the reduction of interparticle interactions. This can be achieved by "dilution" with a solvent, and numerous examples of self-assembled structures of these types have been reported for rodlike [50] and disclike nanoparticles [51]. An alternative method for reducing interparticle interactions and to increase fluidity in the system is to coat the nanoparticle with a suitable organic sheath, and attempts of this type are discussed below.

In the case of pseudospherical polyhedral NPs, their lack of a naturally preferred orientation becomes a hurdle for their arrangement into LC phases, although regular 2-D arrangement into hexagonal lattices has been observed for simple monolayer-protected NPs on surfaces [52]. Whilst theoretical treatments predict that Au NPs coated with simple linear thiols should exhibit spontaneous asymmetry at low temperatures or in solution [53–55], simple NP-hybrids of this type are yet to exhibit LC phases. Therefore, it is clear that the ligands must impart sufficient anisotropy into the hybrid system to force the pseudospherical particles to form ordered self-assembled structures whilst maintaining a fluid state. Thus, parameters such as the orientational flexibility and mobility of the ligands on the NP surface are just as important as their chemical structure, or in other words, their mesogenic character.

### Synthesis of liquid-crystal–nanoparticle hybrids

The synthesis of NP hybrids is of course largely determined by the nature of the nanoparticle, and is affected by factors such as the synthetic method used to prepare the inorganic species, the presence of coligands (stabilisers) and the surface chemistry of the particular material under investigation. Furthermore, the relative ease and flexibility of synthesis of the organic ligand(s) means that their design is tailored to the particular surface chemistry and morphology characteristics of the NP under investigation, ensuring a suitable anchoring group and structure-directing groups are present in the molecular structure.

**Figure 2:**
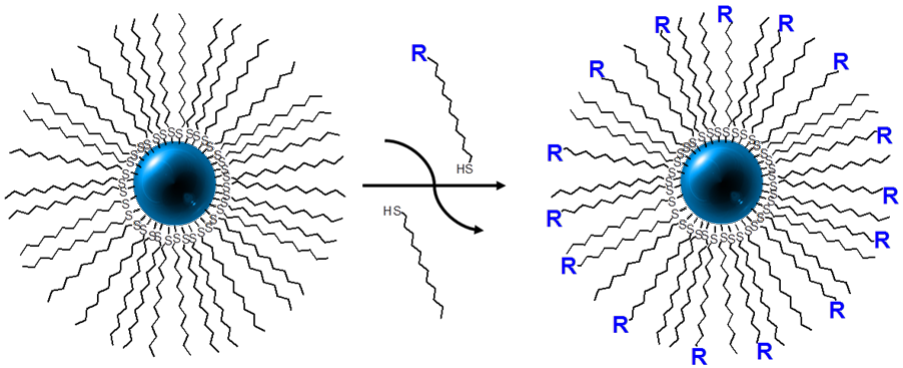
Schematic representation of the solvent-mediated ligand exchange process, illustrated for the partial exchange of thiols at the surface of a noble-metal nanoparticle; R represents a proto-mesogenic moiety.

In the case of Au NPs, which are by far the most extensively investigated, there are two main methods used for the preparation of hybrids; the first being direct synthesis of the NPs in the presence of the ligand of interest by using a modified Brust–Schiffrin procedure [56], and the other being a two-step process in which the NPs are first synthesised with a protective layer (e.g., a simple alkanethiol), followed by solvent-mediated ligand exchange to give the desired product (Figure 2) [12]. The first method gives a NP surface coated with exclusively (but not necessarily completely) the ligand of interest, whereas the second method invariably gives products with mixed ligand coatings. For NPs other than gold, the typical method is to prepare the NPs and then to add the ligand of interest (especially for metal oxides), or to perform a solvent-mediated ligand exchange in a similar manner to that described for the Au NPs above.

Regardless of the method of synthesis used, the purity of the final isolated sample is of course of utmost importance, since the presence of excess LC or non-mesogenic coligands could interfere with LC determination and give spurious results. Purification of the hybrids is typically undertaken through repeated precipitation, through centrifugation steps, or through chromatography techniques such as gel-permeation chromatography (GPC), or through a combination of these methods. Product purity and ligand-grafting rates and ratios can be determined through the use of techniques such as NMR spectroscopy, thermal analysis (DSC/TGA), elemental analysis, and other spectroscopic techniques (e.g., UV–vis).

At this point, it is worth making a note of the nomenclature used in this review. Ligand families are assigned a unique number, followed, where necessary, by numerals indicating the length of any alkyl chains appended to the molecule, which can be cross-referenced with the ligand charts shown throughout the text. Hybrids are represented by the general chemical formula of the NP followed by the ligand involved, separated by an "@" symbol. So, for example, an Au NP coated with ligand **1** is represented as "**Au@1**". If an alkylthiol of chain length *x* is present as a coligand on the surface of the nanoparticle, then nomenclature of the form "**Au@C_x_/1**" is used when necessary.

### Nanoparticles coated by rodlike proto-mesogenic ligands: End-on attachment

The most common and perhaps the most obvious method for inducing LC phases in NP hybrids is to introduce a suitably functionalised LC ligand onto the surface of the particle of interest. In so doing, it is hoped that the self-assembling and mesogenic potential of the ligand will be transferred to the hybrid product. Therefore, given their importance in liquid-crystal science, it is not surprising that rodlike or calamitic mesogenic molecules are the most commonly employed ligands to date in the preparation of liquid-crystalline nanoparticles. Importantly, all of the ligands chosen to date have a flexible spacer between the rigid rodlike segment of the molecule and the functional group used to attach the ligand to the NP surface, and in the case of pseudospherical NPs, the ligands usually display sizes commensurate with the size of the inorganic centre. Flexibility is an important characteristic that allows the ligands to deform easily, which is particularly true in the case of pseudospherical NPs since they allow the hybrid particles to develop anisotropic shapes, which is crucial for their arrangement into liquid-crystalline phases (except cubic structures).

Despite their apparently ideal shape characteristics, publications reporting on the thermotropic mesophases of suitably functionalised anisotropic NPs are relatively uncommon, even though they were some of the first in the field. These types of materials are of interest for the applications mentioned above, but their added advantage is the possibility to possess anisotropic magnetic, optical and electronic properties [57].

**Figure 3:**
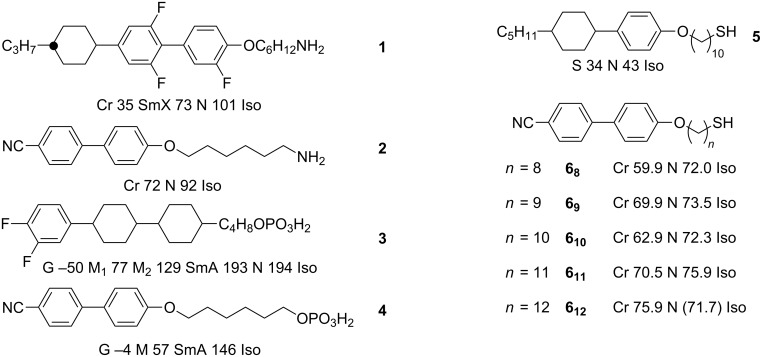
Chemical structures and LC properties of the rodlike ligands discussed in the text.

One of the earliest claims for the preparation of discrete nanoparticles exhibiting thermotropic liquid-crystalline phases involved the synthesis and functionalisation of anisotropic TiO_2_ particles [58]. These nanoparticles were synthesised by using a sol–gel method in a variety of shapes and sizes, and subsequently functionalised by mixing them directly with an equal mass of amino-functionalised mesogenic ligands **1** and **2** (Figure 3).

Interestingly, highly anisotropic **TiO_2_@1** hybrids several hundred nanometres in length and up to ~30 nm in width melted at 48 °C to give a series of optical textures up to 250 °C. X-ray analysis of the mesophase at 70, 90 and 100 °C indicated the existence of periodic particle interactions at an interval of 33.2 nm, which corresponded to the width of the NPs plus the surrounding organic sheath, and the authors concluded that the mesophase possessed a quasi-nematic 1-D order in the direction of the long axis of the acicular particles. The mesophase behaviour was lost for hybrids prepared with **1** and NPs with lower aspect ratios and a deliberately prepared polydisperse sample, and furthermore, none of the hybrids prepared with the cyanobiphenyl based amine **2** exhibited any mesophases.

In an extension of this work, mesophase behaviour was reported for systems involving α-Fe_2_O_3_ NPs coated with mesogenic ligands containing a phosphonic acid anchoring group [59]. The NPs were prepared by using a sol–gel method, and grafting of the ligands was undertaken with 1:1 and 1:2 ligand/NP mass ratios. Mesogenic behaviour was observed for spindle-shaped **α-Fe_2_O_3_@3** 1:2 hybrids, with those prepared with NPs ~300 nm long and ~50 nm wide at 90 °C displaying a marbled texture under polarised optical microscopy (POM). SAXS experiments indicated the existence of periodic particle interactions at intervals of 49.7 and 46.5 nm respectively, which corresponded to the width of the NPs with a layer of ligand molecules on the surface. As was observed for **TiO_2_@1**, these results indicated that the hybrids of **α-Fe_2_O_3_@3** exhibited a quasi-nematic one dimensional order in the direction of the long axis of the spindlelike NPs, which was lost upon the use of a polydisperse **α-Fe_2_O_3_** sample. Importantly, 1:2 and 1:1 **α-Fe_2_O_3_@3** cuboidal hybrids of ~90 nm size exhibited optically isotropic fluidic phases, and X-ray analysis at 170 °C indicated the presence of a superlattice with a simple cubic LC structure. Thus it was clear that the spindlelike NPs preferred nematic and the cuboidal NPs preferred cubic LC arrangements, respectively, which is an intuitive result highlighting the structure-directing influence of the NP morphology on the resulting mesophase.

**Figure 4:**
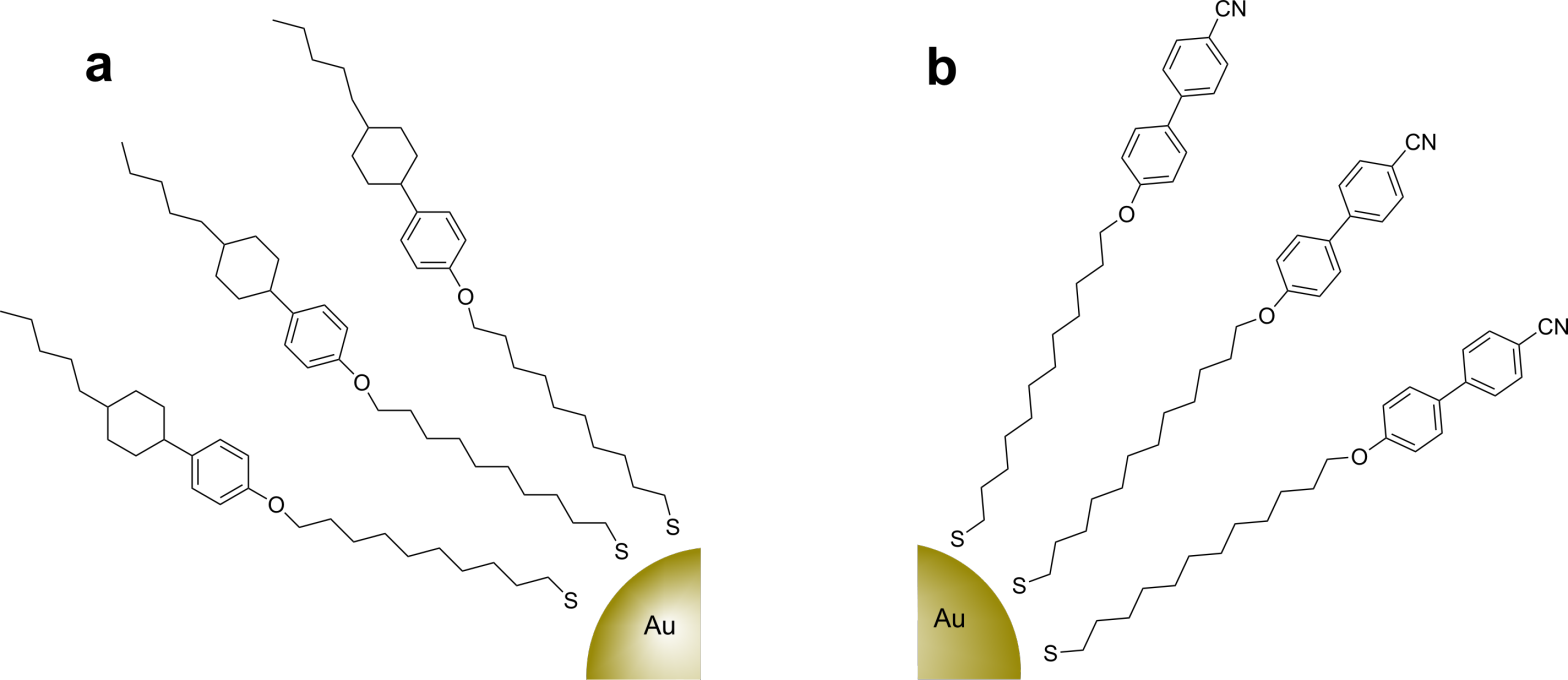
Schematic representation of pseudospherical Au NPs coated exclusively with mesogenic rodlike ligands (a) **Au@5** and (b) **Au@6_12_**.

Pseudospherical NPs are the most common inorganic core used to date in the field of LCNPs, and the first such report involved the direct synthesis of 3 nm Au NPs in the presence of a calamitic (cyclohexyl)phenoxy thiol (**5**, Figure 3) to give a densely packed surface coating (Figure 4a) [60]. The ligand showed both smectic and nematic phases, and the authors claimed that the **Au@5** hybrid displayed higher transition temperatures and a wider mesophase range than the free ligand, as determined from DSC and POM experiments. Unfortunately, no phase assignments were made by diffraction methods, precluding the determination of either the supramolecular interactions of the ligands or the positional arrangement of the NP cores, but this work was important in stimulating further research in the field by a variety of groups.

**Figure 5:**
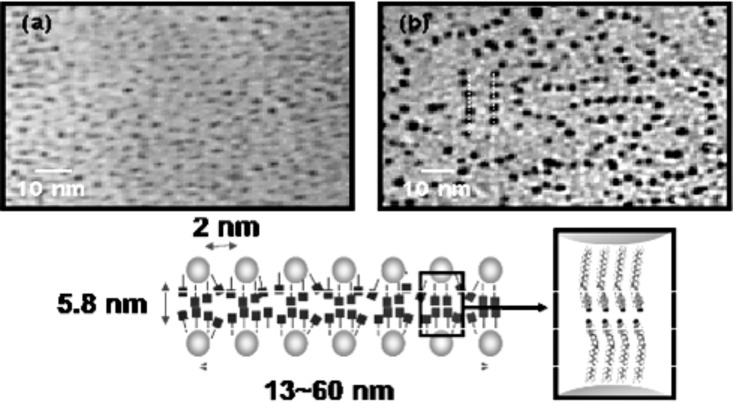
TEM images of **Au@6_12_** (a) before and (b) after thermal treatment. Below: Proposed model of the nanoparticle arrangement within the strings. Reproduced by permission of The Royal Society of Chemistry [61].

Subsequently, a similar procedure was reported in which Au NPs of diameter 2.7 ± 0.5 nm were directly synthesised in the presence of a cyanobiphenyl group appended with an alkylthiol moiety (**6_12_**, Figure 4b) [61]. The thiol ligand exhibited a nematic phase, and the hybrids displayed a mesophase between 110 °C and 130 °C, but no distinct texture could be observed with POM (Table 1). Interestingly, direct observation of the possible arrangement of the NPs was obtained through annealing of a sample in the mesophase range on a TEM grid, followed by quenching to RT (Figure 5). Imaging of the material by TEM revealed 13–60 nm long strings of NPs, with an interstring distance of approximately 5.8 nm (i.e., roughly twice the length of the organic ligands) and an interparticle distance within the strings of 2 nm. These results were corroborated by SAXS analysis, indicating *d* spacings of 6.3 and 2.9 nm.

**Table 1:** Summary of the phase behaviour of the hybrid NPs with rodlike ligands discussed in the text.^a^

NP type	NP size (nm)	Ligand	Phase assignments (Temp. °C)	Ref.

Au	3	**5**	Cr 74 LC 114 Iso	[60]
Au	2.7 ± 0.5	**6_12_**	Cr 110 LC 130 Iso	[61]
Au	2.4	**6_8_**	Meta.: Sm/N (89.7, 106.6; 149.9)	[62]
Au	2.4	**6_9_**	Meta.: Sm/N (85.3; 100.8; 151.6)	[62]
Au	2.4	**6_10_**	Meta.: Sm/N (82.1; 99.2; 151.9)	[62]
Au	2.4	**6_11_**	Meta.: Sm/N (79.8; 92.1; 149.7)	[62]
Au	2.4	**6_12_**	Meta.: Sm/N (85.1; 100.8; 150.5)	[62]

Au	2.0 ± 0.2	**7_9,9_**	Sm 110 Iso	[63]
Au	2.0 ± 0.2	**7_4,4_**	Col Crystal superlattice melts at 150	[63]
Au	2.0 ± 0.2	**7_1,6_**	C_6_:Sm; C_8_: ModSm; C_10_: Col; C_12_:Col; C_18_:NA	[63–64]
Au	2 ± 0.4	**7_2,5_**	C_6_:Sm; C_8_: Sm; C_12_:Col; C_18_:NA	[64]
Au	2 ± 0.4	**8_12,12,12_**	short-range positional order	[64]
Au	2 ± 0.4	**8_0,12,12_**	short-range positional order	[64]
Au	2 ± 0.4	**8_0,8,0_**	C_6_:Sm; C_8_: Sm; C_12_:Sm; C_18_:Sm	[64]
Au	2 ± 0.4	**9**	C_6_:Sm; C_8_: Sm; C_12_:Sm; C_18_:Sm	[64]

^a^LC = unidentified mesophase; Cr = crystal; Sm = smectic; ModSm = modified smectic; N = nematic; Cub = cubic; Col = columnar; Iso = isotropic liquid, Meta = metastable mixture.

The authors argued that the ligands were acting like smectogens in between the strings, but that the distance between NPs within the strings was too small to be caused by end-to-end alignment of the organic moieties, and thus they must be tilted and/or overlapped. This was the first attempt at understanding the phase behaviour of these hybrids by postulating that the ligands were not uniformly disposed about the NP surface, but it was to become a common model used to explain the observed mesophase behaviour of hybrid pseudospherical NPs.

**Figure 6:**
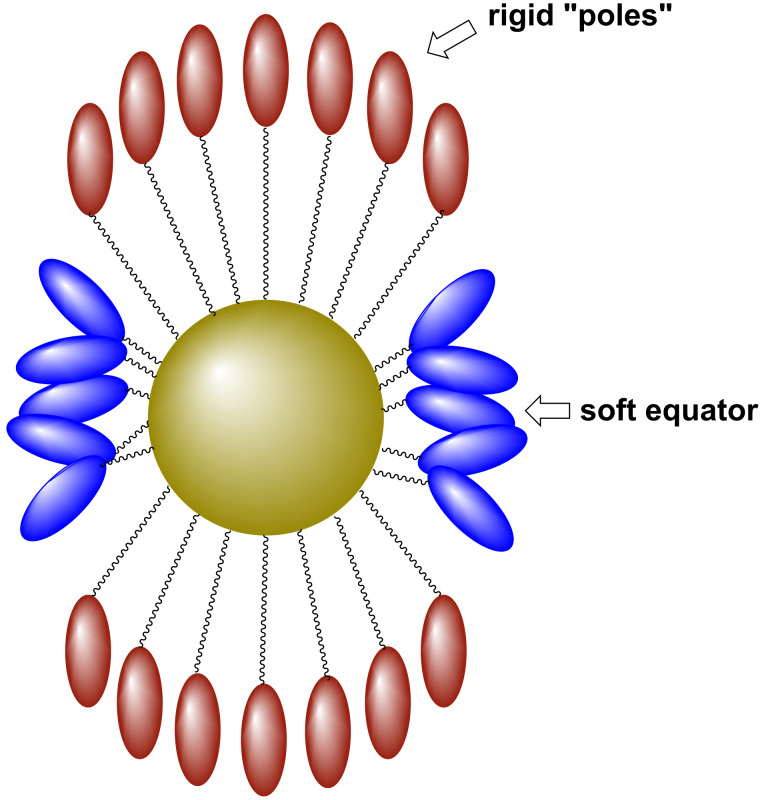
Ligand deformation at the surface of the gold NPs giving rigid "poles" and a soft equator. Such deformations impart the necessary anistropy to the system to impart LC behaviour. Adapted from [62].

Another detailed investigation into the disposition of the ligands about mesogenic Au NPs was reported based on hybrid particles prepared in a two-step process by using a wider suite of mesogenic cyanobiphenyl based thiols [62]. Gold NPs of ~2.4 nm diameter were first synthesised and isolated with a triphenylphosphine protective layer, followed by the addition of thiols **6_8_**_–_**_12_** (Figure 3). The authors estimated the number of ligands attached to the NPs after the exchange process to be ~150–215, which indicated a very dense packing on the surface. Thermal analysis indicated three thermal events for hybrids **Au@6_8_**_–_**_12_** at ~85 °C, ~100 °C and ~150 °C (Table 1), with arguments based on thermochemical values suggesting that the first corresponded well to a melting transition, whereas the third event was reminiscent of a nematic-to-isotropic phase change due to the low enthalpy involved. The X-ray results indicated that the hybrid systems displayed complicated metastable behaviour, with the possible coexistence of more than one phase. In order to rationalise the results, the authors proposed a tactoidal geometry of the NPs, whereby the ligands formed two extended "hard" poles on the nanoparticles and a "soft" equator around the middle, which can be seen as an elaboration of the mechanism proposed previously [61] (Figure 6).

**Figure 7:**
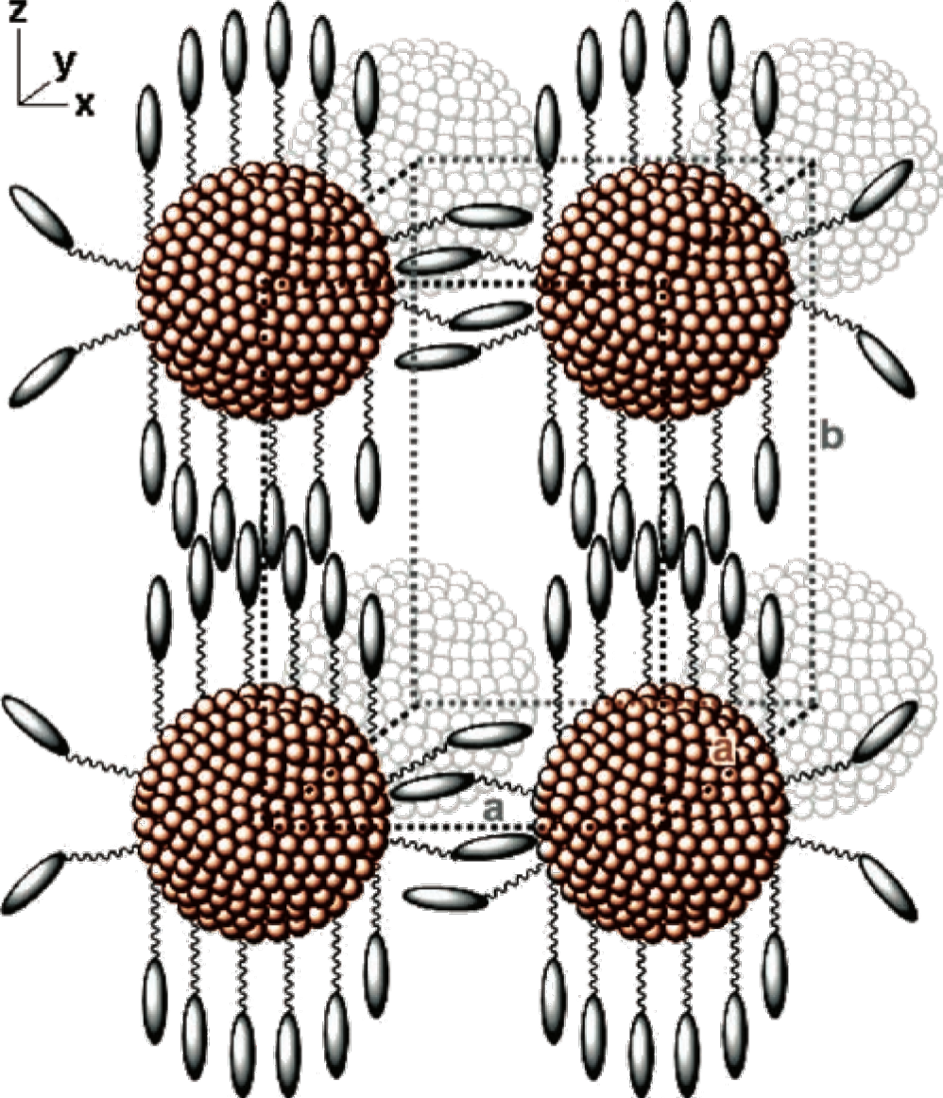
A simplified illustration of the local rectangular arrangement of nanoparticles in a condensed mixed phase. Reprinted with permission from [62]. Copyright 2011 Wiley-VCH Publishers.

Thus a tentative structural model was proposed whereby the NPs exhibit a local rectangular (or hexagonal) arrangement, with a local smectic A_2_ order for the head-to-tail regions, and local smectic A_d_ order for the equators (Figure 7).

**Figure 8:**
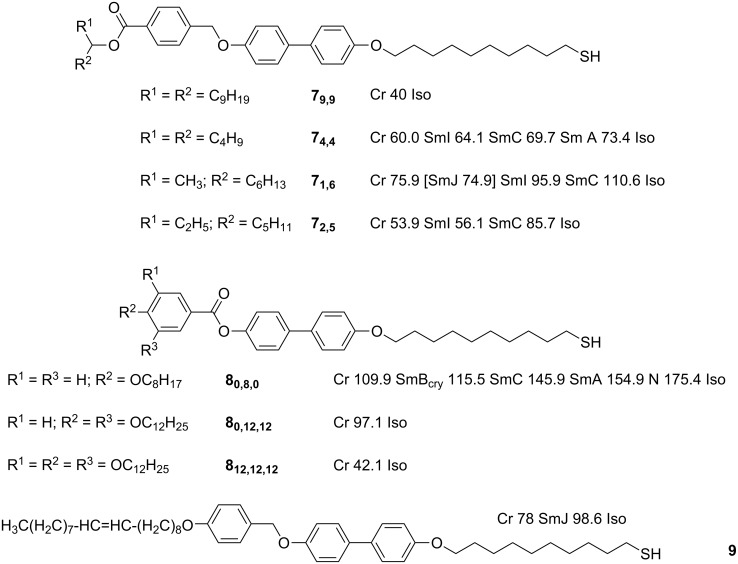
Chemical structures and LC properties of the rodlike ligands discussed in the text.

**Figure 9:**
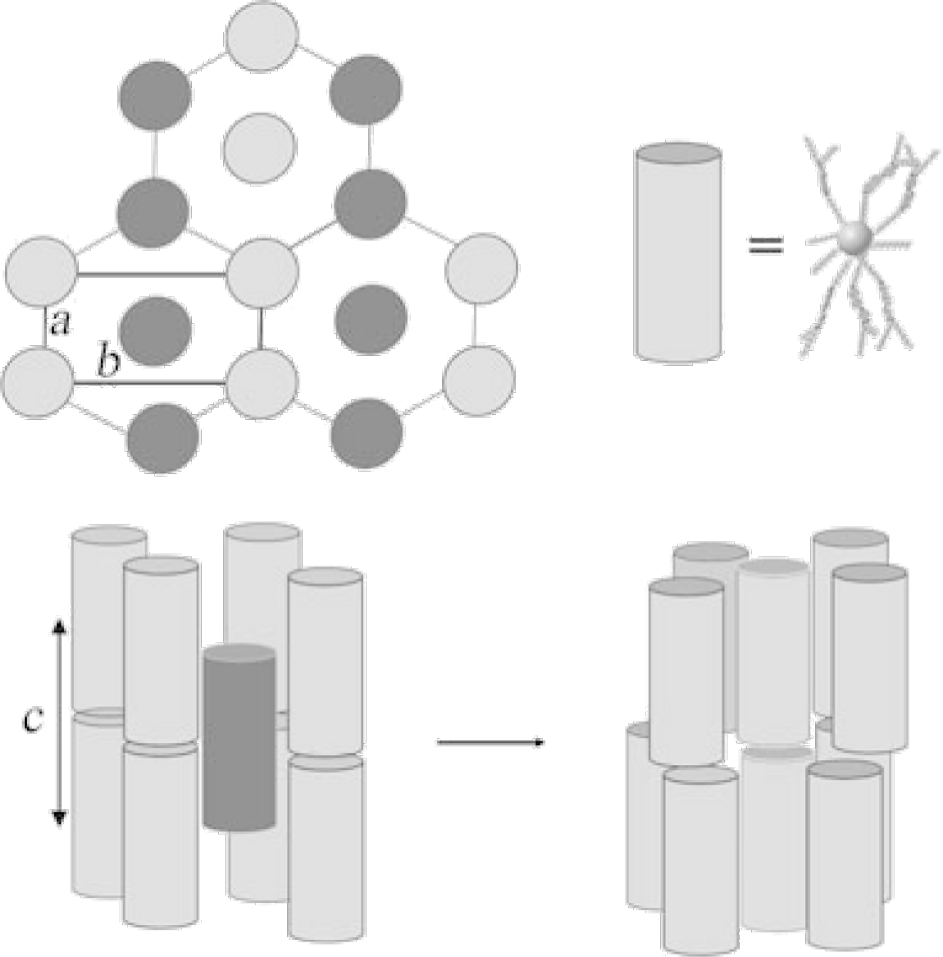
Schematic drawing of the arrangement of nanoparticles in the columnar phase, as viewed from above (top left) and from the side (bottom). The rearrangement of the ligands about the gold NP to form a cylinder is shown (top right). The smectic structure for **Au@C_10_/7_9,9_** (bottom right) can be obtained from the columnar phase for **Au@C_10_/7_4,4_** and **Au@C_10_/7_1,6_** (bottom left) through movement of the particles from the intermediate layer along the column axis and decoupling of the layers. Reprinted with permission from [63]. Copyright 2009 Wiley-VCH Publishers.

In a departure from cyanobiphenyl based ligands, smectic and columnar structures were observed in a large and systematic study for systems involving swallow-tailed rodlike mesogenic thiols **7_9,9_**, **7_4,4_**, **7_1,6_** and **7_2,5_** [63–64] (Figure 8) grafted onto 2.0 ± (0.2–0.4) nm Au NPs in two-step, solvent-mediated exchange processes. The authors noted that, for **Au@C_10_/7_9,9_**, **Au@C_10_/7_4,4_** and **Au@C_10_/7_1,6_** within the experimentally investigated limits of 1:2 and 1:1 mesogen/alkylthiol surface ratios, no differences in phase behaviour were observed [63]. Interesting results were reported for hybrids of ligand **7_9,9_**, a rod-shaped ligand devoid of LC phases, whereby X-ray analysis of **Au@C_10_/7_9,9_** indicated the formation of a smectic A phase (Table 1). An explanation as to how nominally spherical particles could form a smectic A phase was derived from the observation that the thickness of the organic sheath between the layers was greater than the distance between NPs within the layers. This implied that there was some redistribution of the mesogenic groups on the surface towards opposite “poles” of the NPs to give cylinder-shaped hybrids (Figure 9). This mechanism of mesophase induction differs from those discussed previously [61–62] due to the presence of a mixed ligand shell about the metal centre, and thus the importance of ligand migration about the surface in the production of anisotropic hybrids is far more pronounced. It was noted, however, that the organic sublayer was less than twice the length of two rodlike ligands placed end-to-end, and thus the authors concluded that the rodlike ligands remained orientationally and positionally disordered and probably partially interdigitated. The liquidlike nature of the organic sublayer was confirmed by X-ray experiments, and the lack of observable birefringence in the sample was taken as further evidence of the orientational disorder of the molecules inside the organic layer of the smectic materials.

The role of subtle changes to the mesogenic ligand in determining the LC phase of the hybrids was highlighted by the results observed for the mesogenic thiols **7_4,4_**, and **7_1,6_**, which differ from **7_9,9_** only in the length of the alkyl chains on the periphery of the molecule. In the case of **Au@C_10_/7_4,4_** and **Au@C_10_/7_1,6_**, the smectic arrangement observed for **Au@C_10_/7_9,9_** was replaced by columnar structures (Table 1), with the columns organised into body-centred orthorhombic unit cells, and an arrangement very close to being hexagonal. This arrangement is possible by shifting of the particles by *c*/2 along the column axis, which can be readily changed into the smectic phase through another shift of *c*/2, followed by positional decoupling of the layers along the *c* axis (Figure 9).

**Figure 10:**
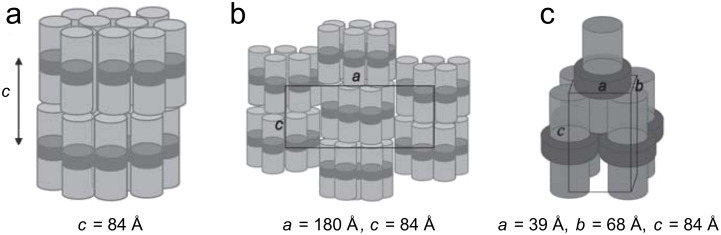
The proposed structural models resulting from ligand migration at the NP surface: (a) Smectic (**Au@C_6_/7_1,6_**) (b) modulated smectic **Au@C_8_/7_1,6_**, (c) columnar (**Au@C_12_/7_1,6_**). Light grey and dark grey indicate the disposition and form of the mesogenic groups and alkylthiols respectively. Reprinted with permission from [64]. Copyright 2010 Wiley-VCH Publishers.

Interestingly, the important role of the alkylthiol coligand (referred to as the “primary grafting layer”) in determining the LC behaviour of these swallow-tailed hybrids was also demonstrated [64] (the effect of the primary grafting layer had been investigated previously for NPs coated with laterally substituted mesogens [65], see below). It was found that the superstructures of the NPs coated with ligands **7_1,6_** and **7_2,5_** were affected by the length of the *n*-alkylthiols in the primary grafting layer. In these cases, particles that were grafted with short hexanethiols in the primary layer (**Au@C_6_/7_1,6_** and **Au@C_6_/7_2,5_**) exhibited smectic phases, whilst increasing the length of the *n*-alkylthiols invoked the formation of modulated smectic and columnar phases (Figure 10). The ligand migration model fits these results well as it explains the formation of the phases based on a geometrical shift from a cylindrical shape for the shorter primary grafting layers, to a more ellipsoidal (or “tri-cylindrical”) shape for those particles with a primary grafting layer composed of longer *n*-alkylthiols (i.e., a “bulge” at the equator of the cylinders).

**Figure 11:**
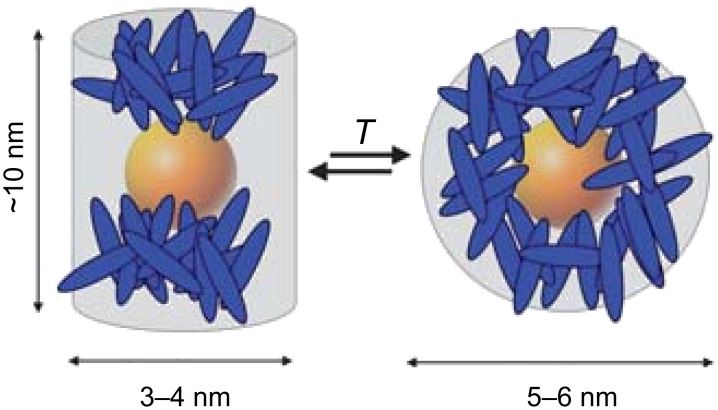
Reversible migration of the surface ligands as a function of temperature (and phase). Only the blue mesogenic ligands are depicted in this image. Reprinted with permission from [64]. Copyright 2010 Wiley-VCH Publishers.

More pronounced changes in the structure of the grafted (proto-)mesogenic ligands produced rather significant changes in the phase behaviour of the resulting hybrids in the case of **Au@8_0,8,0_**, **Au@8_0,12,12_** and **Au@8_12,12,12_** (Table 1)**_._** Whilst the particles coated with proto-mesogenic hemi-phasmidic ligands **8_12,12,12_** and **8_0,12,12_** (Figure 8) displayed only short-range positional order, particles coated with the single-chain mesogenic ligand **8_0,8,0_** (Figure 8) displayed smectic phases, irrespective of the length of the primary grafting layer [64]. These latter results were mirrored for the structurally similar single-chained ligand **9** (Figure 8), which displayed an increase in the interparticle distance within the smectic layers upon an increase in the length of the primary grafting layer. Once again, the orientational disorder of the organic layers was also confirmed by the lack of birefringence in the samples, and the ligand-migration model was used to explain the smectic phases arising from the formation of cylindrical hybrid moieties. This behaviour is reversible upon further heating to the isotropic state, whereby the ligands rearrange to give pseudospherical particles again (Figure 11). The authors argued that the lack of mesogenic behaviour observed for hybrids **Au@8_0,12,12_** and **Au@8_12,12,12_** was due to the large volume required to accommodate the polycatenar ligands at the poles of the NPs, which would create cylinders “pinched” in the middle, and thus unable to arrange themselves into a suitable mesophase structure.

**Figure 12:**
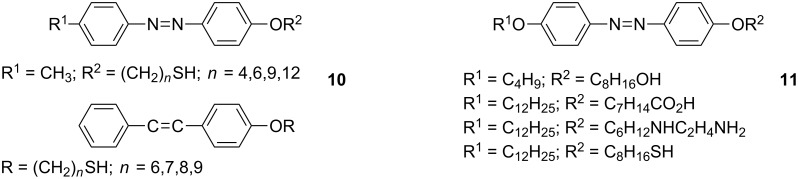
Photochromic and photo-mesogenic rodlike ligands.

It is also worth briefly mentioning a couple of examples in which rodlike mesogenic or proto-mesogenic ligands have been grafted to NPs for studies focused on properties other than liquid crystallinity, in order to emphasise the wider applicability of this approach to nanohybrid science. Fundamental studies into the photochemical activity of lipophilic azobenzene- and stilbene thiols (**10**, Figure 12) on Au NPs surfaces were reported [66], which indicated a chromophore-to-NP distance dependence of the photoisomerisation and photodimerisation efficiency in these systems. Interesting examples of the potential application of this type of approach were shown by the grafting of similar azobenzene ligands **11** (Figure 12) to the surfaces of γ-Fe_2_O_3_ [67] and FePt [68] NPs, which exhibited a photoswitchable change in magnetisation, and which the authors attributed to a change in the electric polarisation at the surface induced by the *cis–trans* isomerisation of the azo moiety upon irradiation, although no indication was given as to whether the hybrids displayed any mesophase behaviour.

### Nanoparticles coated with rodlike proto-mesogenic ligands: Side-on attachment

**Figure 13:**
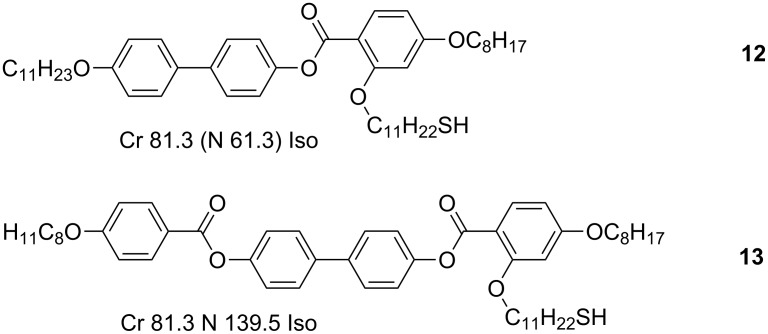
Chemical structures and LC properties of side-on mesogens used to coat NPs.

**Figure 14:**
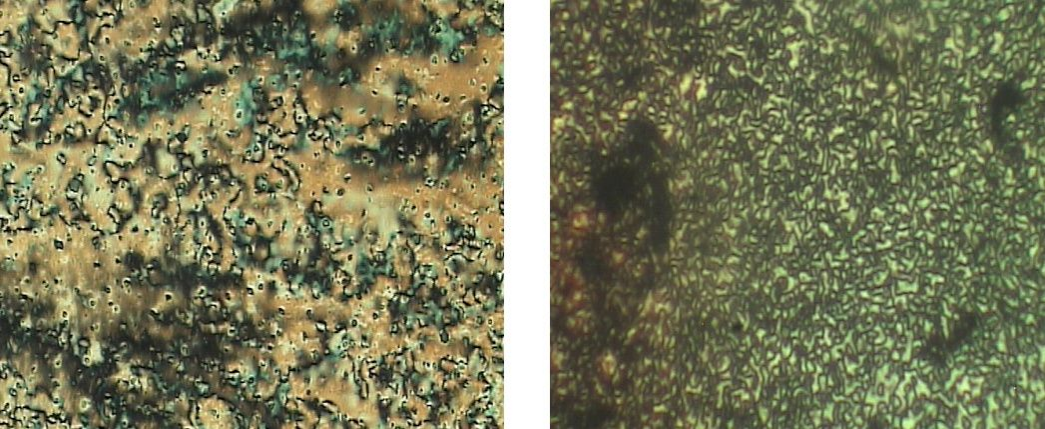
Left: POM image of ligand **12**. Right: POM image of *Schlieren* texture of the hybrid **Au@12**. Reprinted with permission from [69]. Copyright 2006 American Chemical Society.

The first assignment of a nematic phase for a pseudospherical NP was realised through the use of a laterally substituted rodlike mesogenic thiol (**12**, Figure 13) [69]. In this study, Au-NPs of diameters 1.6 ± 0.4 nm were targeted because of their small size and low polydispersity, such that the size variations in the NPs were lower than the maximum size of the mesogenic thiol used. The final hybrids were prepared according to the two-step process, through solvent-mediated ligand exchange of hexanethiol coated NPs with **12**, to give a final surface coverage of 1:1 as determined by ^1^H NMR analysis. Through thermal analysis, POM and miscibility experiments, the existence of the nematic phase was deduced (Table 2), as evidenced by the *Schlieren* and marbled textures observed under crossed polarisers (Figure 14).

**Table 2:** Summary of the phase behaviour of the hybrid NPs with laterally substituted ligands discussed in the text.^a^

NP type	NP size (nm)	Ligand	Phase assignments (Temp. °C)	Ref.

Au	1.6 ± 0.4	**12**	g −3 N 43.8 Iso	[69]
Au	1.7 ± 0.4	**C_6_/13**	Cr 73.1 N^b^ 118.7 Iso	[70]
Au	2.0 ± 0.4	**C_12_/13**	Cr 45.7 N^b^ 126 Iso	[70]
Au	1.7 ± 0.4	**C_6_/13**	Col_r_ 80 Col_h_ 118.7 Iso	[65]
Au	2.0 ± 0.4	**C_12_/13**	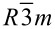 126 Iso	[65]

^a^Cr = crystal; N = nematic; Col_r_ = columnar rectangular; Col_h_ = columnar hexagonal; g = glass; Iso = isotropic liquid. ^b^Provisional nematic assignment, although subsequent experiments indicated a columnar structure.

The success of this approach prompted further work involving a slightly longer rodlike side-on nematogenic ligand (**13**, Figure 13), which was grafted in a solvent-mediated exchange process onto hexane- or dodecanethiol protected Au NPs of 1.7–2.0 nm diameter [70]. NMR spectroscopic analysis of the products indicated that the final alkylthiol:mesogen surface concentrations were ~2:1. Partial coverage of the surface led to thermally unstable hybrids, due to delamination and/or surface reorganisation of the ligands. As reported for the related system above [69], both the hexane- and dodecanethiol coated hybrids **Au@C_6_/13** and **Au@C_12_/13** exhibited mesogenic behaviour, attributed to the formation of an enantiotropic nematic phase, which displayed typical marbled or *Schlieren* textures by POM. Furthermore, the hybrids showed complete miscibility with the neat ligand and its olefinic precursor, which was interpreted as providing further evidence of the existence of a nematic phase. It was noted that the defect textures were formed more easily in the shorter chain hexanethiol system, possibly as a result of greater freedom of movement for the mesogenic groups at the surface. The chain lengths also played a role in the different melting temperatures (45.7 °C for **Au@C_12_/13** versus 73.1 °C for **Au@C_6_/13**, Table 2), with the potential plastifying effect of the longer chains being invoked as a possible explanation. Particles coated exclusively with **13** showed only a glass transition, and this was attributed to the lack of orientational mobility of the mesogenic groups at the surface of the NPs.

**Figure 15:**
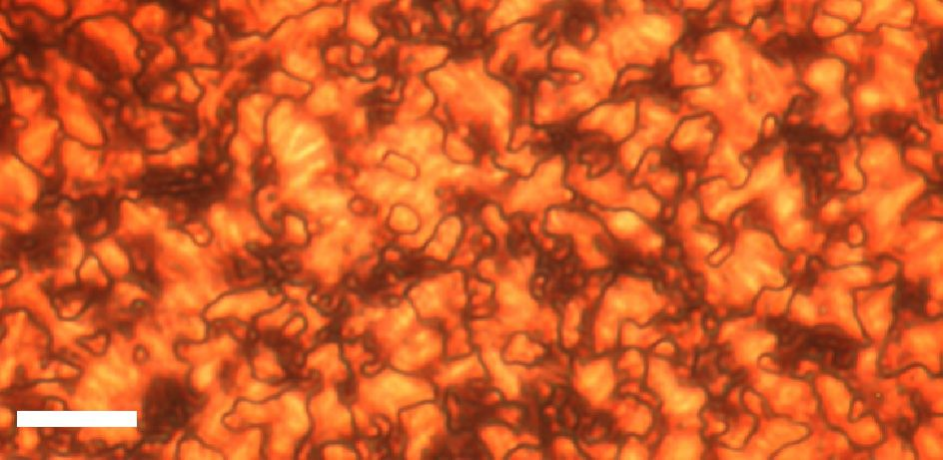
Threaded nematic texture of **Au@ C_12_/13** as observed by POM at RT. Scale bar = 10 μm. Reprinted with permission from [65]. Copyright 2009 Wiley-VCH Publishers.

In a subsequent report, a more detailed investigation into the hybrids obtained from ligand **13** and Au NPs was undertaken [65]. Roughly 40% of the protective hexane- or dodecanethiol ligands were exchanged with **13** by using the two-step process, and the hybrids were examined by POM, X-ray and TEM techniques. The hybrids coated with primary dodecane- and hexanethiol layers (**Au@C_12_/13** and **Au@C_6_/13**) displayed optically isotropic phases above 126 °C and 119 °C, respectively (Table 2), but a birefringent threaded texture typical of a nematic phase below these temperatures (Figure 15).

**Figure 16:**
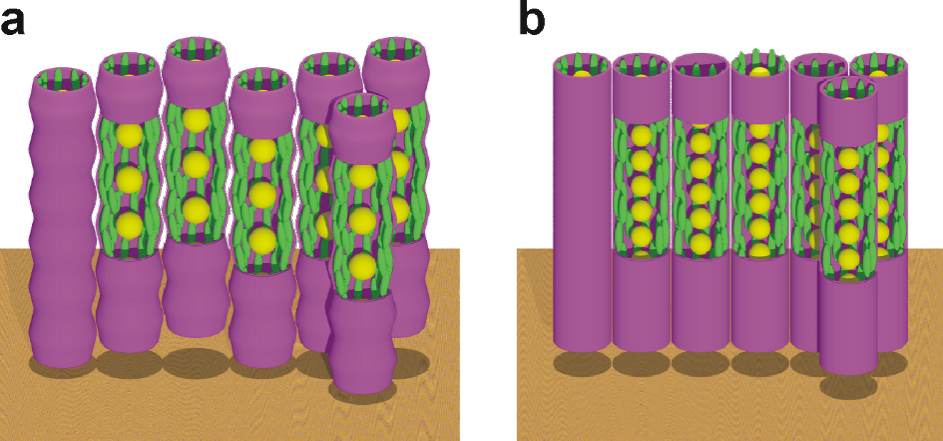
Schematic representation of the gold NP columnar structures. (a) Rhombohedral phase in **Au@C_12_/13** and (b) hexagonal columnar phase in **Au@C_6_/13**. Gold NPs = yellow, ligands = green. Reprinted with permission from [65]. Copyright 2009 Wiley-VCH Publishers.

**Figure 17:**
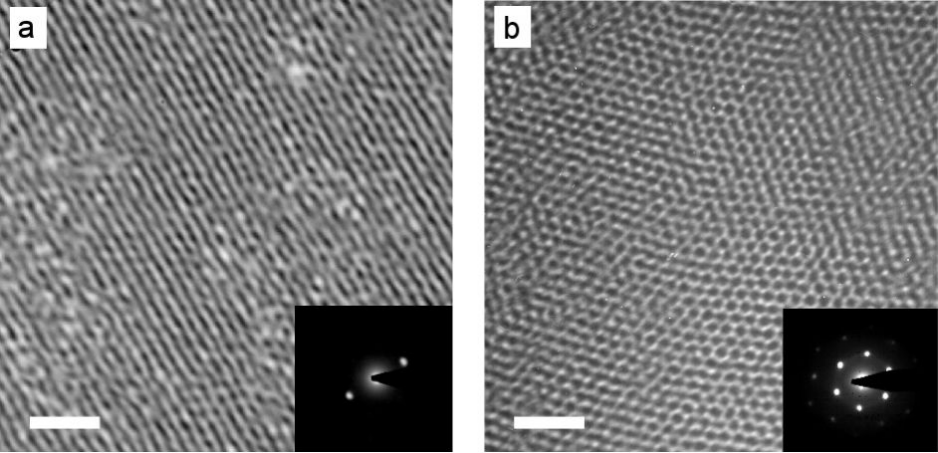
TEM images of thin films of the 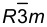 phase of **Au@C_12_/13** recorded with the beam (a) parallel to the 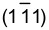 plane and (b) parallel to the [001] axis. Scale bar = 20 nm. Insets show the corresponding electron-diffraction patterns. Reprinted with permission from [65]. Copyright 2009 Wiley-VCH Publishers.

Interestingly, X-ray analysis of **Au@C_12_/13** (Figure 16a) and **Au@C_6_/13** (Figure 16b) indicated that the particles were arranged into columns in both cases. In the case of **Au@C_12_/13** the GISAXS pattern indicated a rhombohedral lattice (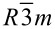 space group), the columns being arranged in a triangular lattice, with the three columns displaced along the *z*-axis by 0, *c*/3 and 2*c*/3 (Figure 16a). The 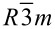 structure of the LC phase was also confirmed by TEM observation, whereby a triangular lattice is observed when viewing the phase down the [001] axis, and stripes are observed when the sample is viewed perpendicular to the 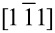 direction (Figure 17).

Between 30 and 80 °C, **Au@C_6_/13** exhibited a 2-D rectangular lattice with a plane group of *c*2*mm*, whereas above 90 °C the lattice was 2-D hexagonal, *p*6*mm*, with the transition arising from shrinking in the *a* direction. Thus, there was 2-D lateral long-range ordering of the columns in the case of **Au@C_6_/13**, whereas **Au@C_12_/13** displayed evidence of 3-D long-range ordering. The structural model indicated that both **Au@C_12_/13** and **Au@C_6_/13** were comprised of NP columns, with the surrounding mesogens axially aligned in the column direction. Despite these results, however, the optical textures and defects observed with POM were distinctly characteristic of a nematic phase, and thus implied the absence of any positional order.

This study clearly highlighted the important role of the connectivity and morphology of the ligands in orienting the pseudospherical NPs [71] into the columnar structures observed. Columnar ordering of pseudospherical cores functionalised with laterally substituted mesogens has been observed previously for liquid-crystalline silsesquioxanes [72–74], indicating that this self-assembly motif may prove to be quite general. Importantly, this work demonstrated that the interparticle spacings within the columns could be modulated not only by the combined effects of the mesogenic and alkylthiol coligands (16.5 Å for **Au@C_12_/13**, and 4.8 Å for **Au@C_6_/13**), but also during the rectangular to hexagonal phase change for **Au@C_6_/13**. These results are of interest due to the fact that, by controlling and modulating the interparticle distances through ligand design and thermally induced phase changes, one can also anticipate the possibility of controlling the electronic and magnetic properties of nanoparticle assemblies that depend on interparticle separations [7,14].

### Nanoparticles coated with bent-core ligands

**Figure 18:**
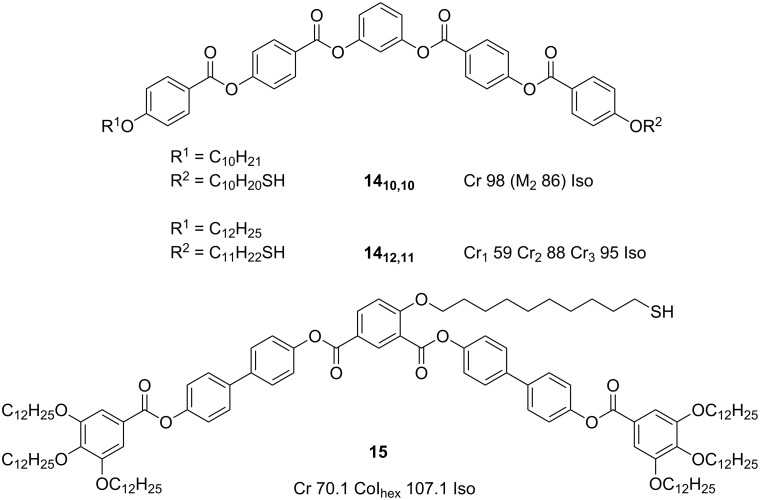
Chemical structures and mesogenic properties of bent-core proto-mesogenic ligands used to coat NPs.

Bent-core mesogens display a bend in their molecular structure (Figure 18), which leads to new packing motifs and provides the possibility of forming mesophases exhibiting polar order and chiral superstructures, even in the absence of any intrinsic chirality. These interesting and unique properties have made them attractive targets in research and development, both academically and for potential applications such as electro- and nonlinear optical devices and photonics [34,75]. This interest has extended to the new field of liquid-crystalline nanoparticles, and some preliminary investigations into the integration of bent-core mesogens into new NP hybrids have been reported.

An early report investigated the grafting of bent-core ligands **14_10,10_** and **14_12,11_** (Figure 18) to hexanethiol coated Au NPs with diameters of 2.8 ± 0.6 and 2.5 ± 0.5 nm by TEM (3.9 ± 1.5 nm and 2.4 ± 0.8 nm by XRD), respectively, in a two-step process to give a 1:1 mixed monolayer coverage [76]. Bent-core ligand **14_10,10_** displayed a monotropic LC phase whilst **14_12,11_** was not mesomorphic. Unfortunately, the hybrids did not display any LC phases, though they could be dispersed into some selected bent-core LC hosts, which showed evidence of a reduction in the electro-optical response time, τ, but a relatively unaffected spontaneous polarisation, *P*. The lack of mesophase behaviour for the pure hybrids was attributed, at least in part, to the high transition temperatures of the bent-core ligands essentially preventing any mesophase development before the onset of sample decomposition through thiol desorption. In another report, despite the success in producing mesophases with Au NPs with a variety of rodlike mesogens, side-on grafting of polycatenar bent-core mesogen **15** (Figure 18) onto decanethiol protected 2.0 ± 0.2 nm Au NPs also failed to produce any LC phases, although X-ray analysis indicated that the hybrid may exhibit some short range fcc or distorted icosahedral structure arising from the presence of cybotactic groups up to 200 °C [63].

### Nanoparticles coated with proto-dendritic ligands

One of the major design factors that must be considered when preparing hybrid NPs for potential LC applications is the relative sizes of the core and the organic shell. It stands to reason that as the thickness ratio between the ligand and the NP increases, the shape-directing properties of the deformable organic shell become more dominant, and thus this parameter could be a powerful tool in the preparation of LCNPs. Increasing the size ratio of the organic component can be achieved by either decreasing the size of the core particle, and/or increasing the thickness of the surrounding molecules, with the latter approach finding success for example in the case of polymer-coated NP hybrids [38–41]. Dendrimers and dendrons offer some advantages in this context, due to the ability to exert a high degree of control over their structure and composition, with their size readily increased through additional generational growth. These properties have led to their exploitation in a number of applications, from catalysis and molecular electronics to nanomedicine [77], and their use in liquid crystal applications is relatively well established [31,78].

**Figure 19:**
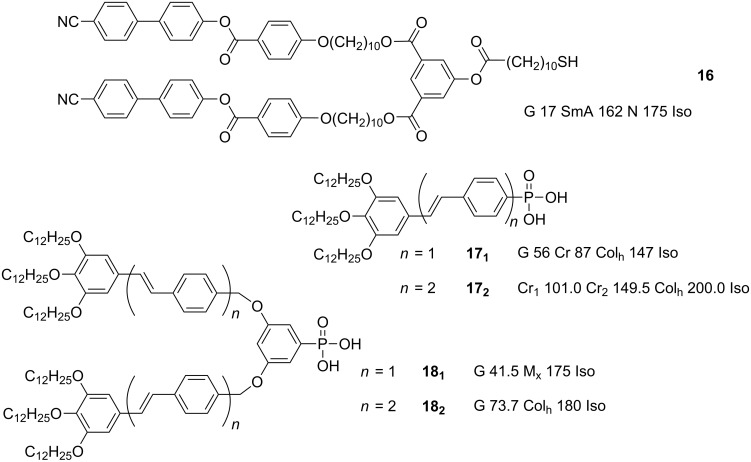
Chemical structures and mesogenic properties of dendritic and proto-dendritic ligands used to coat NPs.

**Figure 20:**
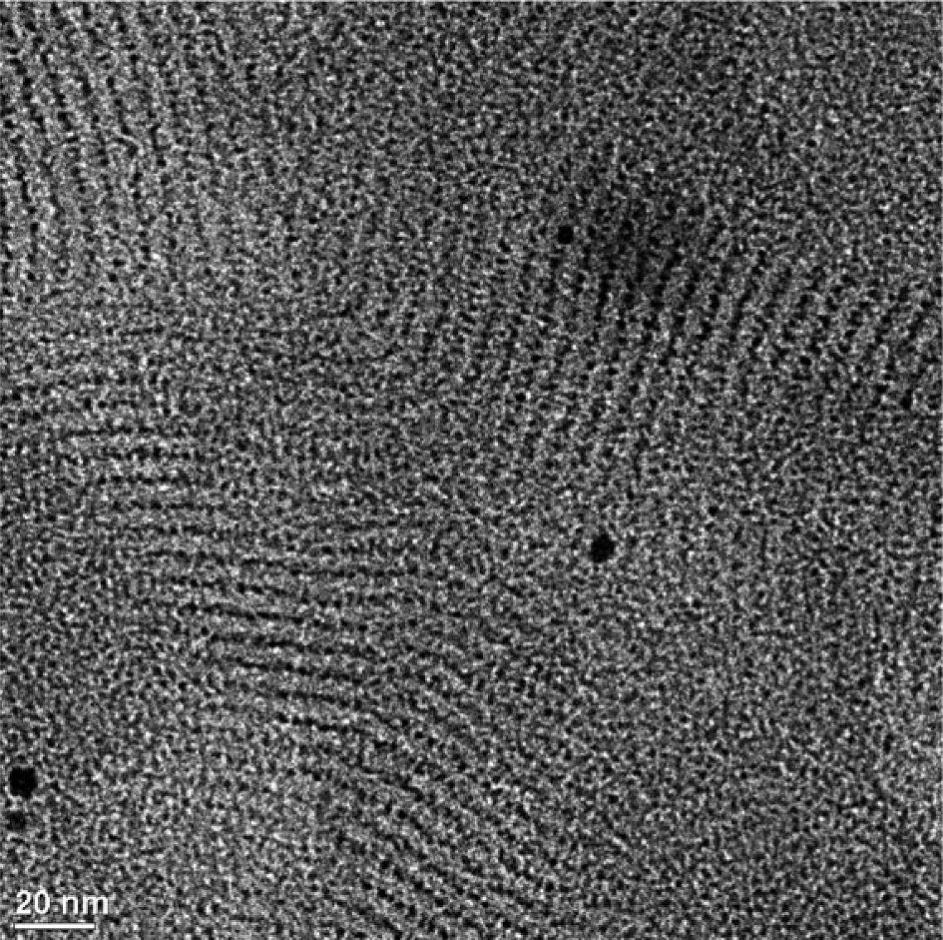
TEM image showing the arrangement of the hybrid NPs **Au@16** into regularly spaced rows. Reprinted with permission from [79]. Copyright 2008 Wiley-VCH Publishers.

Recognising this potential, a study was reported [79] in which a series of liquid-crystalline and non-liquid-crystalline dendrons were prepared containing thiol or disulfide groups within their structures. Hybrid Au NP materials were prepared with cyanobiphenyl terminated dendron **16** (Figure 19) by using both direct and solvent-mediated ligand exchange methods. The direct method produced very small Au NPs of diameter 1.2 ± 0.4 nm coated exclusively with dendron **16**. Using the two-step method, hybrid Au NPs with diameters of 1.7 ± 0.4 nm were prepared, with a surface concentration of approximately 40% of the mesogenic dendron on the surface, as determined by ^1^H NMR spectroscopy. Unfortunately, both of the hybrids with 100 and 40% loading of **16** failed to display any mesomorphic properties, although the fully covered hybrids were able to form highly ordered structures on carbon-coated copper grids (Figure 20). Interestingly, the NPs organised themselves into stripes or rows, with a gap of 6.5 nm between the rows (i.e., 1.3 times the length of compound **16**), but smaller than 1 nm within any given row. This organisation was far less pronounced in the sample containing 40% of **16** on the NP surface. Whilst these compounds did not exhibit any LC phases, the results of the surface arrangement of these hybrids are useful for considering how the ligands are disposed about the metallic centre, and how they interact with each other. The authors proposed that the cyanobiphenyl groups must be interdigitated between the rows, giving a smectic-type arrangement on the surface, with the extremely short interparticle distance within the rows implying that the hybrids are highly anisotropic in shape, either from ligand migration on the surface to the poles of the NPs, and/or through a significant orientational bias of the ligands away from the particle equators towards the poles.

In a departure from the work on gold NPs, an investigation into the functionalisation of pseudospherical ferrite NPs with luminescent prodendritic ligands was undertaken in order to investigate the thermal, magnetic and luminescent properties of the resulting hybrids [80]. The protodendritic oligo(phenylene vinylene) derived ligands included phosphonic acid functional groups (**17** and **18**, Figure 19) for anchoring onto the surface of the NPs. The ferrite nanoparticles were prepared by using a coprecipitation method, followed by a hydrothermal growth process to give cuboidal NPs of 39 ± 5 nm width. These particles were then functionalised with the OPV ligands by direct grafting in solution, followed by purification by magnetic separation or centrifugation, to give surface coverages of 100% for the polycatenar G0 ligands **17_1_** and **18_1_** and 67% and 88% for the G1 ligands **17_2_** and **18_2_**, respectively, as determined by TGA and UV–vis spectral analyses. Whilst the dendronised NPs maintained their intrinsic magnetic properties, none of them displayed any mesophases, and the simplest explanation for this begins with the large size discrepancy between the ligands and the NPs, combined with the potentially unfavourable morphology of the cuboidal NPs (although large size discrepancies between ligand and NP did not appear to inhibit LC formation in the case of large anisotropic, including cuboidal, NPs previously [58–59], see above). In this case, the rigidity of the ligands coupled to the lack of a flexible spacer between the ligand and NP surface probably also played a role in inhibiting LC-phase formation, although similar systems involving the direct attachment of rigid rodlike ligands without a flexible spacer to Au NPs allowed their incorporation into a nematic LC host [81]. Ligands **17** and **18** would be unable to distort to any significant extent, and furthermore, they would not have been of sufficient bulk to induce anisotropy to the hybrids, serving merely as potential "lubricants" to reduce interparticle interactions through their long terminal alkyl chains.

At this point, it is worth noting that much of the work reported on liquid-crystalline NPs has involved the use of mesogenic ligands grafted to the NP of choice in order to induce mesophases in the resulting hybrids. An alternative holistic approach, whereby none of the constituent parts are mesomorphic in isolation, but which display mesomorphic phases upon their combination into one hybrid system has also been reported [82]. This publication was the first to report on dendrimer-functionalised gold NPs that exhibited a thermotropic cubic phase and 2-D hexagonal arrangement on a surface.

**Figure 21:**
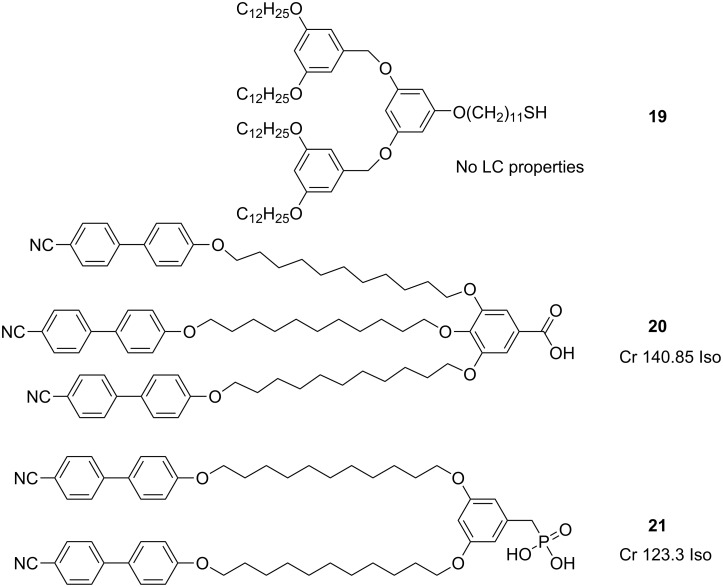
Chemical structures and mesogenic properties of dendritic and proto-dendritic ligands used to coat NPs.

Dodecanethiol coated Au NPs of diameter 2.1 ± 0.5 nm were partially coated with dendron **19** (Figure 21) by solvent-mediated ligand exchange to give a final surface coverage of ~4:5 dendron to dodecanethiol. This represents a rather dense surface coverage, corresponding to an average chain cross-sectional area of *A*_Chain_ = 18.1 ± 0.5 Å^2^, which is below the value of 21.4 Å^2^ expected for alkanethiols on flat gold surfaces. This discrepancy was rationalised as being due to the ability of the thiols to adsorb also at the edges between the flat polyhedral faces of the nanoparticle surface, thus increasing the effective loading relative to a macroscopic planar system.

The dendritic part was decoupled from the NP surface by a dodecyl chain spacer to allow flexibility and to maintain the conical dendron structure at a distance from the NP. This was thought to be advantageous for enhancing the interparticle interactions and to aid in the stabilisation of the mesophase, with the peripheral alkyl chains included to ensure that the system was fluid. The overall design of the hybrid resembled that of spherical micellar structures, which are good candidates for the formation of cubic arrangements in the mesophase.

**Figure 22:**
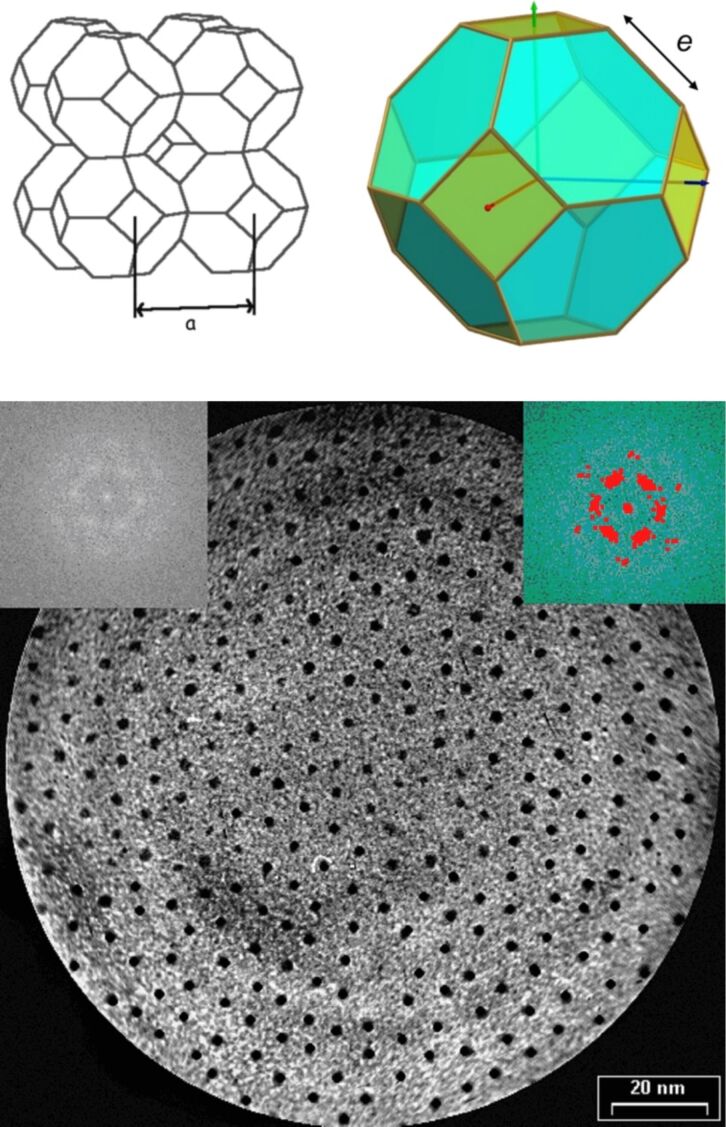
Top left: Body-centred (I) cubic lattice of 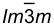 symmetry composed of truncated octahedrons. Top right: The truncated octahedron—a semiregular Archimedean tetradecahedron. Bottom: Self-organisation in a hexagonal lattice as observed by TEM. Reprinted with permission from [82]. Copyright 2007 Wiley-VCH Publishers.

Differential scanning calorimetric and SAXS analysis of the hybrid indicated a melting transition at −12.2 °C (heating)/−16.5 °C (cooling), and that the particles possessed liquidlike order between −10 °C and 60 °C (Table 3), which changed to a cubic-lattice arrangement of space group 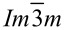 at higher temperatures (i.e., the packing arrangement of the material involved the cubic arrangement of a single type of pseudospherical (truncated octahedral) hybrid particle, one at the corners and one at the centre of the unit cell; Figure 22). This "inverse-melting" thermal behaviour is a relatively uncommon phenomenon, but it has been observed in various chemical systems [83] and is known as "re-entrance" [84].

**Table 3:** Summary of the phase behaviour of the hybrid NPs with dendritic and proto-dendritic ligands discussed in the text.

NP type	NP size (nm)	Ligand	Phase assignments (Temp. °C)^a^	Ref.

Au	2.1 ± 0.5	**19**	Cr −12.2 Iso 70–80 Cub 180 Dec	[82]
Fe_2_O_3_	3.3 ± 0.7	**20**	g 40–60 N 180.2 Iso	[85]
Fe_2_O_3_	3.3 ± 0.7	**21**	g 30–50 N 174.2 Iso	[85]

^a^Cr = crystal; N = nematic; Cub = cubic; g = glass; Iso = isotropic liquid; Dec = decomposition.

The dendronised nanoparticles spontaneously self-assembled into a hexagonal lattice on the TEM grid: The mean distances between first and second neighbours were about 7.6 and 13.7 nm, respectively, in very good agreement with the NP diameter and the dendritic shell (Figure 22). This system also displayed ferromagnetism between 1.8 K and 400 K, and an unusually slow magnetic relaxation, which reaches a maximum at around the transition temperature between the isotropic liquid and the cubic phase [86]. These results are of interest from the standpoint of information storage applications, and could lead to the development of new materials with novel magnetic properties.

In another report in which a similar approach was utilised [85] the synthesis and functionalisation of pseudospherical magnetic ferrite NPs [87] with pro-mesogenic ligands was described. The NPs were synthesised by the thermal decomposition of iron oleate complexes at high temperature in high-boiling-point solvent to give products of 3.3 ± 0.7 nm diameter. The cyanobiphenyl terminated ligands **20** and **21** (Figure 21) with carboxylic acid and phosphonic acid anchoring groups, respectively, were grafted onto the surface of the NPs by means of a ligand-exchange process to displace some of the adsorbed oleate protective layer. Thermogravimetric analysis was used as a means of determining the surface loading of the ligands, and the results indicated that the phosphonic acid derivative (100 ± 10 **21** : 50 ± 10 OA) was three times more efficient at adsorbing to the surface than the carboxylic acid derivative (30 ± 10 **20** : 95 ± 10 OA). Ligands **20** and **21** were both non-mesogenic, directly melting into isotropic liquids at 140.5 °C and 123.2 °C respectively, whereas the hybrids exhibited higher clearing temperatures of ~180 °C (**Fe_2_O_3_@20**) and 174 °C (**Fe_2_O_3_@21**) (Table 3). Upon heating, the samples developed a low birefringent and fluid texture, which could not be assigned to any specific mesophase, and below 50–60 °C these mesophases froze into a glassy state.

**Figure 23:**
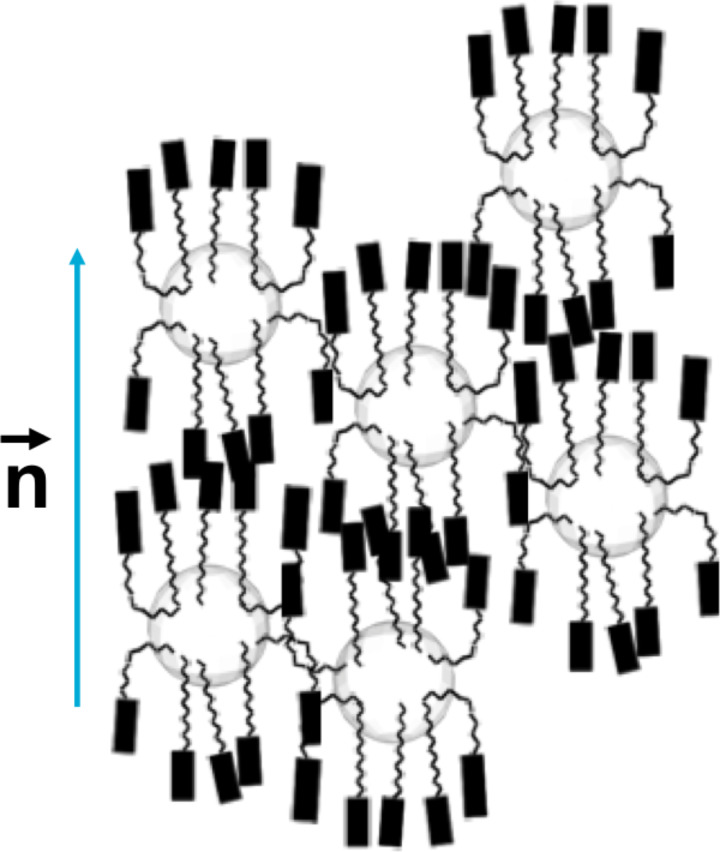
Model proposed for the organisation of the hybrids within the quasi-nematic mesophase. Reprinted with permission from [85]. Copyright 2010 Wiley-VCH Publishers.

X-ray analysis of the materials upon heating indicated average interparticle periodicities of 7.5 ± 0.5 nm (**Fe_2_O_3_@20**) and 6.9 ± 0.1 nm (**Fe_2_O_3_@21**), which corresponded well with the sizes of the NPs with their organic coatings. The correlation length for **Fe_2_O_3_@21** was found to be about three times that of **Fe_2_O_3_@20**, which the authors argued was caused by the increased cohesion between the hybrid NPs due to more efficient interdigitation of the cyanobiphenyl groups. The observation of a second, weak diffusion occurring at around 1.5 nm (**NP@20**) and 1.8 nm (**NP@21**) was assigned to weakly correlated cybotactic clusters, as in classical nematic phases. Thus, the mesophase was assigned as a quasi-nematic phase consisting of a quasi-regular isotropic distribution of the NP nuclei within the organic matrix characterised by a local nematic order (Figure 23). This view of the packing was also confirmed by TEM imaging.

**Figure 24:**
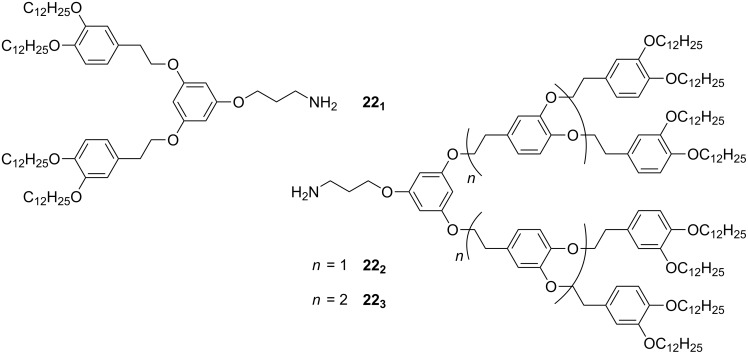
Mesogenic dendrons used to coat Au NPs.

A recent report [88] describes the grafting of dendrons **22_1_**_–_**_3_** (Figure 24) to carboxylic acid functionalised Au NPs of diameters 5.9 ± 0.6, 6.8 ± 0.7 and 6.8 ± 0.8 nm through a surface amidation reaction. Interestingly, the hybrids only displayed 2-D hexagonal organisation (TEM) when coated with the second generation dendron **22_2_**, with a random arrangement observed for NPs coated with **22_1_** and **22_3_**. This ordering behaviour was broadly consistent in 3-D as well, whereby hybrids coated with **22_1_** and **22_3_** showed no order, but the hybrid of **22_2_** with Au NPs of 6.8 ± 0.7 nm diameter exhibited a primitive cubic phase after annealing of the sample.

### Nanoparticles coated with discotic proto-mesogenic ligands

Discotic mesogens are those which display one molecular axis considerably shorter than the other two, and they are extensively investigated [89–90] due to their potential use in a variety of high-technology applications [89], perhaps most notably for opto-electronic devices [91] and organic electronics [92]. The basis for much of this interest lies in their inherent ability to self-organise into columnar structures, which exhibit directional properties, such as electrical conduction along the column axis.

**Figure 25:**
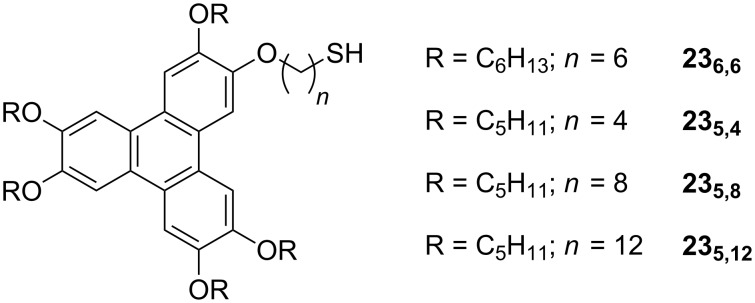
Chemical structures of the discotic mesogenic ligands used to coat NPs.

Au NPs of 2.4 nm average diameter coated with triphenylene based mesogenic thiols **23_6,6_** (Figure 25) were prepared by using the direct method to give a surface exclusively coated with the discotic ligands [93]. These materials did not display mesomorphism in the neat state, but were readily dispersed into the columnar phase of triphenylene discotic liquid crystals without disruption of the nature of the mesophase. Furthermore, thin films of **Au@23_6,6_** displayed 2-D hexagonal ordering on TEM grids, with interparticle distances less than twice the length of the organic moieties suggesting that the ligands at the surface were intercalated to some extent.

**Figure 26:**
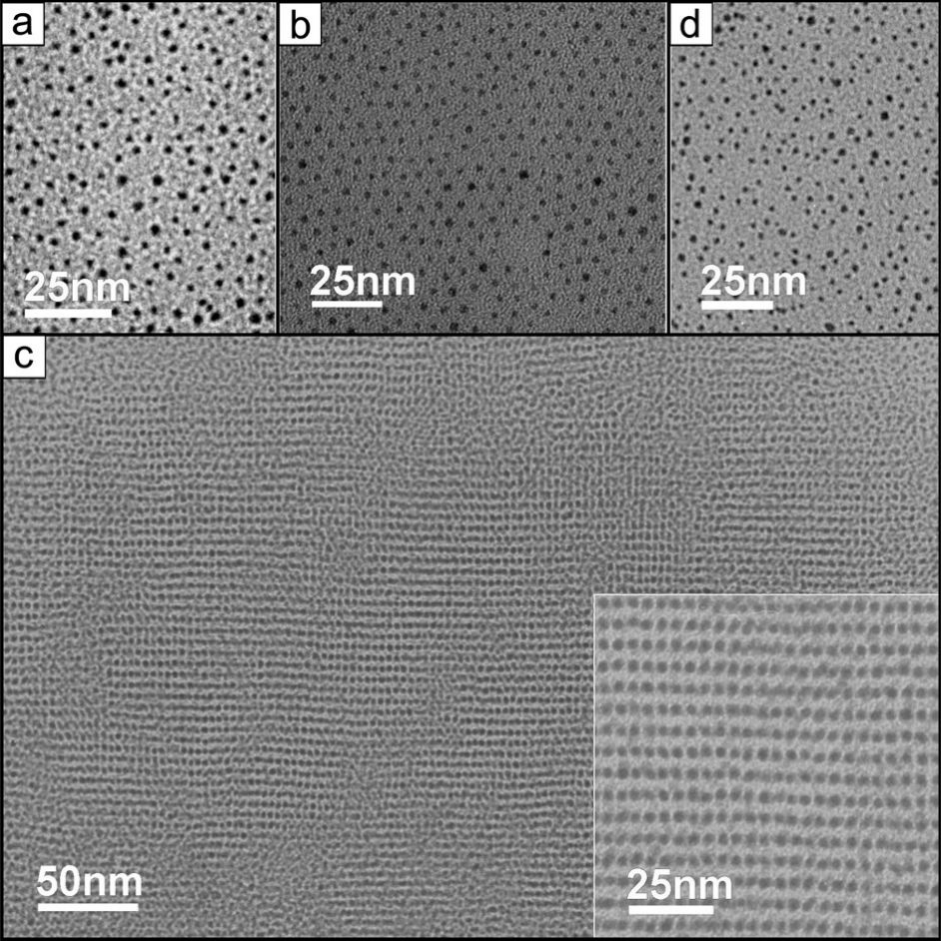
TEM images of **Au@23_5,12_** prepared from aged solutions stood for 10 days in solutions of (a) 1:1 MeOH/toluene (disordered), (b) 2:1 MeOH/toluene (hcp), (c) 3:1 MeOH/toluene (stripelike; inset is an expansion), (d) 4:1 MeOH toluene (disordered). Reprinted with permission from [94]. Copyright 2007 American Chemical Society.

In another investigation into ligands of this type [94], although no mention was made of their liquid-crystalline properties, it was shown that NPs coated with triphenylenes **23_5,4_**, **23_5,8_** and **23_5,12_** (Figure 25) were able to self-assemble into beautiful 2-D structures with hexagonal, squarelike and stripelike arrangements as a function of both the length of the tethering group and the solvent polarity (Figure 26). These results bode well for the use of these discotic materials in NP self-organisation, and it appears only a matter of time before LC phases are observed for discrete hybrids of this type.

## Conclusion

Whilst it is clear that the field of liquid-crystalline nanoparticles is still in its infancy, the exciting results reported to date indicate that these new materials are viable, with a few early trends emerging from the literature. By coating NPs with suitably designed ligands it is possible to soften the interparticle potentials and introduce sufficient anisotropy to allow the hybrids to self-organise into ordered fluid phases. From this starting point, it is unsurprising to find that the mesogenic properties of these hybrid NP systems depend on a synergy between the nature of the grafted ligand and the size and shape of the NP under investigation.

Starting with the core of the hybrid, one observes that the morphology of the nanoparticle is of supreme importance in determining the mesomorphic properties of the systems reported to date, though further investigation is warranted in order to clarify as to what effect the chemical composition of the NP could have on the phase behaviour of the materials. The size and shape are critical parameters as expected, with the results reported also indicating that highly polydisperse samples display no discernable mesophase behaviour, which is consistent with theoretical treatments [95–96]. This is particularly apparent for systems in which the organic ligand is small relative to the size of the NP, the clearest examples of this are seen for highly anisotropic NPs, which form nematic phases upon suitable functionalisation, whereas cuboidal NPs form simple cubic phases. Nevertheless, the presence of an anisotropic NP functionalised with a mesogenic ligand is not sufficient to guarantee the formation of a mesophase, as it has been shown that the aspect ratio may play an important role in determining the ability of the hybrid materials to self-assemble. This can be understood by considering the packing constraints acting upon these hard particles, whereby LC ordering would be impeded by the void volumes that are present between particles due to their local curvature. Approaches involving the use of thick, deformable organic coatings may provide an avenue towards the realisation of mesophase behaviour in these systems, with dendrons and polymers being the most likely candidates for this role.

**Figure 27:**
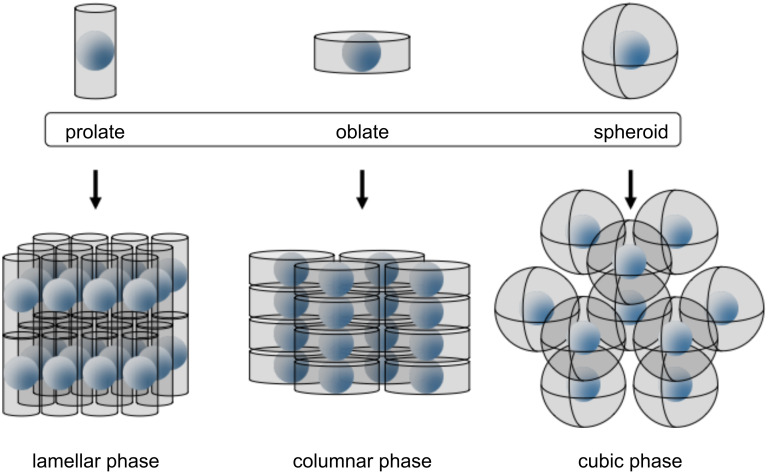
Some of the various hybrid geometries and packing motifs possible upon ligand grafting to the surface of a pseudospherical NP. The role of the ligand(s) in migrating and deforming at the surface is crucial in providing these structures.

Of course, design arguments based solely on the shape and size of the hybrid components do not allow one to gain a clear overall picture of the dynamic nature of LCNPs. It is also necessary to consider the nature of the ligand itself, be it mesogenic or otherwise, in the context of its shape, anisotropy, flexibility, mobility and its surface concentration. Based on the limited results available, it is not possible to state definitively whether coating of a nanoparticle exclusively with the mesogenic ligand of interest promotes or inhibits mesophase formation, as examples are available showing both behaviours. Neither is it possible to guarantee LC-phase formation in the presence of a mixture of mesogenic and non-mesogenic coligands. However, what does become clear, at least in the case of pseudospherical NPs, is that the surface species must have sufficient flexibility and mobility in order to either migrate about the surface of the NP, and/or orientationally distort themselves such that the overall hybrid can obtain the anisotropic shape necessary for the formation of mesophases. Ligand reorganisation akin to the formation of separate surface domains [97] appears to be a dominant mechanism in the case of mixed-ligand systems, whereas ligand deformation into hard poles and a soft equatorial region has been proposed for systems involving a single mesogenic ligand on the surface. These mechanisms highlight the pivotal role of the ligand in influencing the structure of the mesophase, allowing the pseudospherical nanoparticles to be arranged into a variety of phases, such as nematics, smectics, columnar and cubics (Figure 27). Importantly, it is apparent that mesogenic ligands are not always necessary for inducing mesogenic properties in the NP hybrids. The LC phases reported for systems involving non-mesogenic ligand precursors indicate that it should be possible to consider the design of these LC hybrid materials in a more holistic manner, whereby the whole is more than simply just the sum of its parts.

At this point, it is worth mentioning a word of caution regarding the characterisation of these new materials. The phase behaviour of typical "simple" organic LCs can often be characterised with a certain degree of confidence using a mixture of thermal analysis and POM, and with mixing tests with compounds exhibiting the same phase used as a confirmation. Early results have indicated that such a strategy could be quite misleading in some NP hybrid systems, with the POM textures (or lack thereof) observed for the hybrids bearing no relation to those observed for simple organic LCs exhibiting the same phase (e.g., nematic, smectic etc.). Similarly, phase assignments made through mixing experiments should also be treated with caution. It therefore becomes imperative that researchers in this field endeavour to characterise their materials fully using diffraction techniques, complemented by the array of other traditional methods (e.g., TEM, POM).

Encouragingly, it is clear that the field of LCNPs is wide open for chemists to design and synthesise new hybrids, and it only seems a matter of time before we will see not only a wider variety of phases observed, but also new applications for these novel materials. By controlling the spatial assembly of NPs into ordered structures and combining the processability and defect tolerance of the LC state, the structural and functional versatility of the organic coating, and the unique magnetic, optical and electrical properties (including synergistic collective behaviours and the emergence of new physical properties [98–100]) of NPs, liquid-crystalline nanoparticles are poised to address some of the exciting challenges of this century. The future looks bright for the preparation of addressable assemblies of multifunctional hybrids, including high-density recording media [101–102], single-electron microelectronic [103] and charge-transport devices [4], nanoscale plasmon waveguides [104] and metamaterials [62,105]. The field of nanotechnology will only advance whilst scientists have a variety of methods available to them for the production of the nanostructures that they desire, and LCNPs show tremendous promise in adding an important tool for the fabrication of exciting new materials.
